# Prior Direct Oral Anticoagulant Therapy is Related to Small Infarct Volume and No Major Artery Occlusion in Patients With Stroke and Non‐Valvular Atrial Fibrillation

**DOI:** 10.1161/JAHA.118.009507

**Published:** 2018-08-23

**Authors:** Yuki Sakamoto, Seiji Okubo, Tetsuro Sekine, Chikako Nito, Satoshi Suda, Noriko Matsumoto, Yasuhiro Nishiyama, Junya Aoki, Takashi Shimoyama, Takuya Kanamaru, Kentaro Suzuki, Masahiro Mishina, Kazumi Kimura

**Affiliations:** ^1^ Department of Neurological Science Graduate School of Medicine Nippon Medical School Tokyo Japan; ^2^ Department of Radiology Graduate School of Medicine Nippon Medical School Tokyo Japan; ^3^ Department of Neuro‐Pathophysiological Imaging Graduate School of Medicine Nippon Medical School Tokyo Japan

**Keywords:** anticoagulant, arterial occlusion, atrial fibrillation, direct oral anticoagulant, infarct volume, non‐valvular atrial fibrillation, occlusion, Cerebrovascular Disease/Stroke, Magnetic Resonance Imaging (MRI)

## Abstract

**Background:**

The aims of the present study were to investigate the relationships between prior direct oral anticoagulant (DOAC) therapy and infarct volume and the site of arterial occlusion in patients with acute ischemic stroke and non‐valvular atrial fibrillation.

**Methods and Results:**

From March 2011 through November 2016, consecutive patients with acute ischemic stroke in the middle cerebral artery territory and non‐valvular atrial fibrillation were recruited. The infarct volume was assessed semi‐automatically using initial diffusion‐weighted imaging, and the arterial occlusion site was evaluated on magnetic resonance angiography. The effect of prior DOAC treatment on the site of arterial occlusion was assessed by multivariate ordinal logistic regression analysis. A total of 330 patients (149 women; median age 79 [quartiles 71–86] years; median National Institutes of Health Stroke Scale score 11 [4–21]) were enrolled. Of these, 239 were on no anticoagulant, 40 were undertreated with a vitamin K antagonist (VKA), 22 were sufficiently treated with VKA (PT‐INR ≥1.6), and 29 were on a DOAC before the acute ischemic stroke. The infarct volume on admission differed among the groups (median 14.5 [2.0–59.8] cm^3^ in patients with no anticoagulation, 24.8 [2.1–63.0] in undertreated VKA, 1.3 [0.3–13.5] in sufficient VKA, and 2.3 [0.5–21.0] in DOAC,* P*=0.001). Multivariate analysis showed that prior DOAC treatment was independently and negatively associated with more proximal artery occlusion (odds ratio [OR] 0.34, *P*=0.015), compared with no anticoagulant.

**Conclusions:**

DOAC treatment before the event was associated with smaller infarct volume and decreased risk of greater proximal artery occlusion in acute ischemic stroke patients with non‐valvular atrial fibrillation, compared with no anticoagulation.

**Clinical Perspective**

**What Is New?**

- The present study showed that direct oral anticoagulant medication before the stroke was related to smaller infarct volume and decreased risk of more proximal arterial occlusion in patients with non‐valvular atrial fibrillation and ischemic stroke, compared with no anticoagulation or undertreated vitamin K antagonist therapy.

**What Are the Clinical Implications?**

- Anticoagulant therapy with direct oral anticoagulants for patients with non‐valvular atrial fibrillation could reduce stroke severity, when patients develop ischemic stroke.

## Introduction

Patients with atrial fibrillation (AF) often develop severe ischemic stroke and have poor outcomes after stroke,^1^, ^2^ even with thrombolytic therapy.^3^ Anticoagulant treatment with a vitamin K antagonist (VKA) has been proven to reduce the incidence of ischemic stroke for patients with AF.^4^ Moreover, VKA therapy, especially with sufficient anticoagulation, has been reported to reduce the severity and improve clinical outcomes when patients with AF suffer ischemic stroke, compared with those on no anticoagulation.^5^, ^6^, ^7^, ^8^ Decreasing the clot size, to avoid major artery occlusion and therefore small infarct volume is considered the main mechanism by which VKA therapy alleviates initial symptoms in acute ischemic stroke patients.^9^, ^10^, ^11^, ^12^

Direct oral anticoagulant (DOAC) therapy has been shown to reduce the risk of ischemic stroke in patients with non‐valvular AF (NVAF) as well as VKA.^13^, ^14^, ^15^, ^16^ Anticoagulant treatment with DOACs was considered to contribute to consistent anticoagulation than with VKA. Therefore, DOACs also have a potential to reduce the initial severity of ischemic stroke when patients taking DOACs suffer a stroke, at least as well as sufficient VKA treatment.^17^ However, the effect of prior DOAC therapy on major artery occlusion or infarct volume in acute stroke is not well known. The aims of the present study were to investigate the relationships between prior DOAC therapy and infarct volume and the site of arterial occlusion in patients with acute ischemic stroke and NVAF.

## Methods

The data, analytic methods, and study materials will not be made available to other researchers for purposes of reproducing the results or replicating the procedure because the original data included the patients’ personal information.

### Patients

From March 2011 through November 2016, consecutive acute stroke patients (<7 days from onset) with NVAF who were admitted to our stroke unit and fulfilled the following criteria were retrospectively enrolled from the prospective registry: (1) underwent magnetic resonance imaging (MRI) examinations including diffusion‐weighted imaging (DWI) and time‐of‐flight magnetic resonance angiography on admission; and (2) developed ischemic stroke in the middle cerebral artery (MCA) territory confirmed on initial DWI with compatible acute neurological deficits. Patients with contraindications to MRI (eg, cardiac pacemakers) or valvular AF were excluded. This study was approved by the institutional ethics committee. Written informed consent was obtained from all patients or their next‐of‐kin.

### Clinical Characteristics

Clinical background characteristics, including sex, age, cardiovascular risk factors, and past medical histories, were recorded on admission. Cardiovascular risk factors were defined as: (1) hypertension: history of using antihypertensive agents, systolic blood pressure ≥140 mm Hg, or diastolic blood pressure ≥90 mm Hg before or ≥2 weeks after stroke onset; (2) diabetes mellitus: use of hypoglycemic agents, random glucose level ≥200 mg/dL, or glycosylated hemoglobin ≥6.5% on admission; (3) hyperlipidemia: use of antihyperlipidemic agents, or a serum total cholesterol level ≥220 mg/dL; and (4) current smoker. The prestroke CHADS_2_ or CHA_2_DS_2_‐VASc score was calculated for each patient based on the published guideline^18^ and patients’ clinical characteristics. However, aortic plaque was not assessed as a component of the score, because transesophageal echocardiography was performed for only about one quarter of the patients included in the present study. The index stroke was not counted as “history of ischemic stroke”. Stroke severity was assessed using the National Institutes of Health Stroke Scale (NIHSS) and functional status was evaluated with modified Rankin scale (mRS). Routine blood biochemistry examinations were performed on admission.

### Neuroimaging

MRI studies including DWI and time‐of‐flight magnetic resonance angiography were performed on admission using a commercially available echo planar instrument operating at 1.5 T (Echelon Oval, Hitachi Medical Systems, Tokyo, Japan). DWI was obtained using the following parameters: TR/TE, 6000/65 ms; b‐values, 0 and 1000 s/mm^2^; field of view, 24 cm; acquisition matrix, 128×128; and slice thickness, 4.5 mm, with a 2.5‐mm intersection gap. The infarct volume was assessed semi‐automatically using DWI on admission and image analysis software (3D Slicer, http://www.slicer.org). 3D Slicer, built through support from the National Institutes of Health, is a free open‐source extensible software application for medical image computing and visualization.^19^ The patients’ Digital Imaging and Communications in Medicine data were imported, and a hyperintense lesion on DWI was semi‐automatically outlined on each slice using segmentation tools within 3D slicer. The hyperintense area (cm^2^) was multiplied by the slice thickness plus intersection gap, resulting in a volume measurement (cm^3^). The site of arterial occlusion was determined on initial magnetic resonance angiography: at the internal carotid artery; at the middle cerebral artery horizontal segment; at the middle cerebral artery insular segment; and no identifiable occlusion.

### Statistical Analysis

The patients were divided into 4 groups based on the medication prescribed before the index event: no anticoagulant medication; undertreated with VKA therapy (corresponding to warfarin treatment and prothrombin time‐international normalized ratio [PT‐INR] on admission <2.0 for patients <70 years old and PT‐INR <1.6 for patients ≥70 years old); sufficient VKA therapy (corresponding to warfarin treatment and PT‐INR on admission ≥2.0 for patients <70 years old and PT‐INR ≥1.6 for patients ≥70 years old); and DOAC treatment. The cut‐off level for sufficient VKA anticoagulation was set based on previous studies in Japan^20^, ^21^ and a domestic guideline.^22^ Renal function was assessed by the estimated glomerular filtration rate (eGFR) instead of creatinine clearance on admission. Univariate analyses were performed using the chi‐squared test, Fisher exact test, or the Kruskal–Wallis test, as appropriate. The data are presented as median values (quartiles) or frequencies (%). Next, multivariate ordinal logistic regression analysis was performed to identify independent factors associated with more proximal arterial occlusion. This model allows the outcome variable to have >2 categories and estimates a proportional odds ratio (OR) for each predictor of shifting to a more proximal arterial occlusion category. For example;

1. No occlusion versus middle cerebral artery insular segment, middle cerebral artery horizontal segment, and internal carotid artery occlusion.
2. No occlusion and middle cerebral artery insular segment occlusion versus middle cerebral artery horizontal segment, and internal carotid artery occlusion.
3. No occlusion, middle cerebral artery insular segment, and middle cerebral artery horizontal segment occlusion versus internal carotid artery occlusion.

Sex, age, and all clinical characteristics identified on univariate analyses with *P*<0.1 were entered into the model. CHADS_2_ and CHA_2_DS_2_‐VASc scores were excluded from the model because of duplication of variables, and initial NIHSS, infarct volume, and mRS at discharge were also excluded because these parameters were consequences of, rather than factors associated with, arterial occlusion. The ORs and 95% confidence intervals (CIs) of more proximal arterial occlusion were calculated. All statistical analyses were performed using PASW for Windows version 17.0 software (SPSS Inc, Chicago, IL, USA). Results were considered significant at *P*<0.05.

## Results

Overall, 481 consecutive patients with AF and acute ischemic stroke were admitted to our stroke center during the study period. Of these, 105 patients were excluded because the site of the index stroke was outside the middle cerebral artery territory, 33 were excluded because of no MRI examination on admission, 8 were excluded because of severe motion artifact on DWI, and 5 were excluded because of valvular AF. Therefore, 330 patients (149 women; median age 79 [quartiles 71–86] years and 269 [82%] patients were ≥70 years old; NIHSS score 11 [4–21]; onset to arrival 4.5 [2.0–13.6] hours) were enrolled in the present study. Of these 330 patients, 239 (72%) were on no anticoagulant medication, 40 (12%) were undertreated with VKA, 22 (7%) were on sufficient VKA, and 29 (9%) were on a DOAC (11 patients were administered rivaroxaban, 10 dabigatran, 7 apixaban, and 1 edoxaban) before the index event. Among 239 patients with no anticoagulation, AF was diagnosed before stroke onset in 105 (44%) patients.

Table 1 shows the clinical background characteristics of the included patients. The proportions of patients having a history of dyslipidemia (*P*=0.014), congestive heart failure (*P*=0.002), and systemic embolism including ischemic stroke (*P*<0.001), and the values of the CHADS_2_ (*P*<0.001) and CHA_2_DS_2_‐VASc (*P*<0.001) scores, modified Rankin scale (mRS) score before the index event (*P*=0.028) and discharge (*P*=0.042), NIHSS score on admission (*P*=0.010), aPTT (*P*<0.001), PT‐INR (*P*<0.001), and D‐dimer (*P*<0.001) levels were significantly different among the 4 groups; patients developing ischemic stroke on DOAC treatment tended to have more past history of embolism and higher CHADS_2_ or CHA_2_DS_2_‐VASc scores, but lower NIHSS scores and D‐dimer levels on admission than those without anticoagulant therapy.

**Table 1:** Clinical Background Characteristics of the Included Patients

Variables	Total	No AC	Undertreated VKA	Sufficient VKA	DOAC	*P* Value
	n=330	n=239	n=40	n=22	n=29	
Female sex, n (%)	149 (45)	107 (45)	19 (48)	13 (59)	10 (35)	0.367
Age, y, median (quartiles)	79 (71–86)	79 (71–86)	79 (75–87)	80 (73–88)	77 (72–81)	0.394
Risk factors						
Hypertension, n (%)	213 (65)	149 (63)	28 (70)	15 (68)	21 (72)	0.635
Dyslipidemia, n (%)	110 (34)	72 (30)	19 (49)	12 (55)	7 (24)	0.014
Diabetes mellitus, n (%)	52 (16)	34 (14)	8 (21)	4 (18)	6 (21)	0.643
Current smoker, n (%)	50 (15)	40 (17)	7 (18)	1 (5)	2 (7)	0.234
Congestive heart failure, n (%)	73 (22)	41 (17)	17 (43)	7 (32)	8 (29)	0.002
Prior embolism, n (%)^a^	70 (21)	35 (15)	16 (40)	8 (36)	11 (38)	<0.001
History of vascular disease, n (%)^b^	48 (15)	34 (14)	6 (15)	1 (5)	7 (25)	0.238
CHADS_2_ score, median (quartiles)	2 (1–3)	2 (1–3)	3 (2–4)	3 (1–4)	3 (2–4)	<0.001
CHA_2_DS_2_‐VASc score, median (quartiles)	4 (2–5)	3 (2–5)	5 (3–5)	4 (3–5)	4 (3–5)	<0.001
Preadmission antiplatelet use, n (%)	91 (28)	75 (31)	7 (18)	3 (14)	6 (21)	0.086
Preadmission mRS, median (quartiles)	0 (0–2)	0 (0–0)	0 (0–3)	1 (0–2)	0 (0–3)	0.028
Chronic atrial fibrillation, n (%)	242 (73)	171 (72)	32 (80)	15 (68)	24 (83)	0.405
Onset to arrival, h, median (quartiles)	4.5 (2.0–13.6)	4.5 (2.0–12.0)	3.1 (2.0–10.7)	8.5 (1.9–28.4)	9.2 (1.4–25.6)	0.656
NIHSS score on admission, median (quartiles)	11 (4–21)	12 (4–22)	18 (7–26)	7 (3–15)	8 (2–12)	0.010
Infarct volume at admission, cm^3^, median (quartiles)	11.9 (1.5–46.9)	14.5 (2.0–59.8)	24.8 (2.1–63.0)	1.3 (0.3–13.5)	2.3 (0.5–21.0)	0.001
Arterial occlusion at admission, n (%)						0.012
ICA	51 (16)	35 (15)	12 (30)	1 (5)	3 (10)	
M1	73 (22)	61 (26)	4 (10)	5 (23)	3 (10)	
M2	48 (15)	34 (14)	9 (23)	2 (9)	3 (10)	
No occlusion	158 (48)	109 (46)	15 (38)	14 (64)	20 (69)	
Biochemistry sign at admission, median (quartiles)						
aPTT, s	29.5 (26.6–33.8)	28.7 (26.0–31.8)	29.7 (26.4–34.3)	36.4 (33.4–40.9)	34.6 (29.8–39.3)	<0.001
PT‐INR	1.12 (1.03–1.29)	1.09 (1.01–1.16)	1.34 (1.17–1.46)	2.05 (1.69–2.39)	1.27 (1.13–1.41)	<0.001
Blood glucose, mg/dL	118 (102–144)	118 (103–144)	113 (96–134)	122 (97–181)	131 (103–165)	0.260
Creatinine, mg/dL	0.85 (0.69–1.03)	0.85 (0.68–1.02)	0.79 (0.71–1.07)	0.91 (0.72–1.21)	0.88 (0.72–1.02)	0.694
eGFR, mL/min	60 (47–74)	60 (48–74)	62 (49–74)	52 (34–71)	63 (53–73)	0.335
D‐dimer, μg/mL	1.5 (0.9–3.1)	1.8 (1.0–3.2)	1.5 (1.1–3.2)	1.0 (0.5–1.9)	0.8 (0.5–2.5)	<0.001
Brain natriuretic peptide, pg/mL	204 (118–376)	201 (117–381)	235 (121–400)	244 (75–442)	158 (129–295)	0.900
mRS at discharge, median (quartiles)	3 (2–5)	4 (2–5)	4 (2–5)	2 (1–3)	3 (1–5)	0.042

AC indicates anticoagulant; aPTT, activated partial thromboplastin time; DOAC, direct oral anticoagulant; DWI‐ASPECTS, Alberta Stroke Program early computed tomography score on diffusion‐weighted imaging; eGFR, estimated glomerular filtration rate; ICA, internal carotid artery; M1, middle cerebral artery horizontal segment; M2, middle cerebral artery insular segment; mRS, modified Rankin Scale; NIHSS, National Institutes of Health stroke scale; PT‐INR, prothrombin time‐international normalized ratio; VKA, vitamin K antagonist.

a Including ischemic stroke and systemic embolism.

b Including ischemic heart disease and peripheral artery disease.

The infarct volume on admission was different among the 4 groups (median 14.5 [2.0–59.8] cm^3^ in patients with no anticoagulation, 24.8 [2.1–63.0] cm^3^ in undertreated VKA, 1.3 [0.3–13.5] cm^3^ in sufficient VKA, and 2.3 [0.5–21.0] cm^3^ in DOAC, *P*=0.001); patients with prior sufficient VKA or DOAC tended to have lower infarct volume than those with no anticoagulation or undertreated VKA therapy. The site of arterial occlusion was also different among the 4 groups (*P*=0.012, Figure). Table 2 shows the clinical characteristics of the patients according to the site of arterial occlusion, and variables identified on univariate analyses with *P*<0.1 were entered into the multivariate model as potential confounders. Multivariate ordinal logistic regression analysis showed that prior DOAC therapy was an independent factor associated with decreased risk of more proximal arterial occlusion, compared with no prior anticoagulant therapy (OR 0.34, 95% CI 0.14–0.81, *P*=0.015, Table 3). The proportional odds assumption had been met on the model. Prior DOAC therapy was also independently and negatively associated with more proximal artery obstruction when undertreated VKA therapy was set as a reference in this model (OR 0.22, 95% CI 0.08–0.60, *P*=0.003).

**Figure 1:**
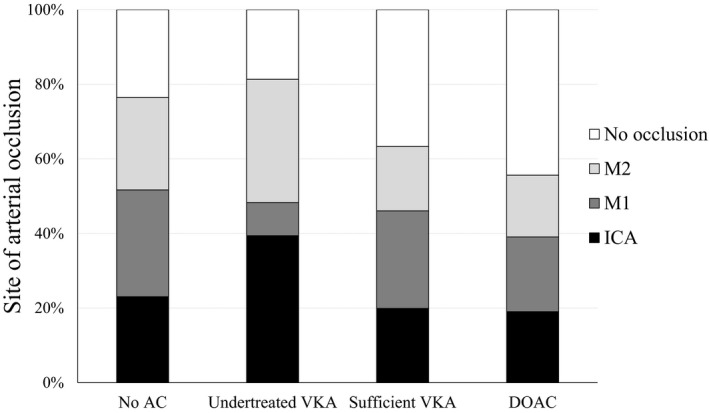
The site of arterial occlusion by anticoagulant status before the index event. The site of arterial occlusion differs among the 4 groups (*P*=0.012). AC indicates anticoagulant; DOAC, direct oral anticoagulant; VKA, vitamin K antagonist.

**Table 2:** Background Characteristics According to the Site of Arterial Occlusion

Variables	ICA	M1	M2	No Occlusion	*P* Value
	n=51	n=73	n=48	n=158	
Female sex, n (%)	29 (57)	35 (48)	21 (44)	64 (41)	0.215
Age, y, median (quartiles)	82 (77–89)	78 (71–85)	80 (72–87)	77 (70–85)	0.014
Risk factors					
Hypertension, n (%)	32 (63)	42 (58)	36 (75)	103 (66)	0.302
Dyslipidemia, n (%)	11 (22)	22 (30)	16 (33)	61 (39)	0.147
Diabetes mellitus, n (%)	11 (22)	13 (18)	7 (15)	21 (13)	0.493
Current smoker, n (%)	5 (10)	12 (16)	10 (21)	23 (15)	0.502
Congestive heart failure, n (%)	19 (37)	16 (22)	14 (29)	24 (15)	0.006
Prior embolism, n (%)^a^	8 (16)	11 (15)	11 (23)	40 (25)	0.263
History of vascular disease, n (%)^b^	9 (18)	8 (11)	8 (17)	23 (15)	0.742
CHADS_2_ score, median (quartiles)	2 (2–3)	2 (1–3)	2 (2–3)	2 (1–3)	0.070
CHA_2_DS_2_‐VASc score, median (quartiles)	4 (3–5)	3 (2–5)	4 (3–5)	4 (2–5)	0.059
Preadmission antiplatelet use, n (%)	16 (31)	21 (29)	11 (23)	43 (27)	0.813
Preadmission mRS, median (quartiles)	0 (0–3)	0 (0–1)	0 (0–2)	0 (0–1)	0.243
Chronic atrial fibrillation, n (%)	40 (78)	55 (75)	37 (77)	110 (70)	0.515
Onset to arrival, h, median (quartiles)	3.0 (2.0–10.0)	3.0 (1.8–7.0)	4.5 (1.6–12.8)	6.6 (2.5–20.1)	0.004
NIHSS score on admission, median (quartiles)	23 (18–26)	19 (14–25)	15 (7–24)	4 (2–10)	<0.001
Infarct volume at admission, cm^3^, median (quartiles)	84.3 (20.5–165.6)	28.7 (7.1–87.1)	17.6 (3.9–51.5)	2.3 (0.4–14.6)	<0.001
Preadmission anticoagulant status, n (%)					0.012
No anticoagulant	35 (15)	61 (26)	34 (14)	109 (46)	
Undertreated VKA	12 (30)	4 (10)	9 (23)	15 (38)	
Sufficient VKA	1 (5)	5 (23)	2 (9)	14 (64)	
DOAC	3 (10)	3 (10)	3 (10)	20 (69)	
Biochemistry sign at admission, median (quartiles)					
aPTT, s	29.5 (27.0–32.8)	29.0 (25.8–33.0)	28.4 (25.8–32.5)	29.8 (27.4–34.7)	0.306
PT‐INR	1.15 (1.06–1.31)	1.12 (1.03–1.23)	1.10 (1.00–1.23)	1.12 (1.04–1.33)	0.275
Blood glucose, mg/dL	127 (110–153)	126 (110–148)	116 (103–144)	111 (99–140)	0.004
Creatinine, mg/dL	0.87 (0.67–1.09)	0.83 (0.68–1.03)	0.84 (0.71–0.97)	0.87 (0.71–1.06)	0.780
eGFR, mL/min	59 (41–73)	60 (51–76)	63 (50–72)	60 (47–73)	0.594
D‐dimer, μg/mL	2.4 (1.5–4.8)	1.9 (1.0–4.1)	1.7 (1.1–2.6)	1.1 (0.7–2.5)	<0.001
Brain natriuretic peptide, pg/mL	338 (195–532)	197 (115–377)	274 (126–454)	163 (92–288)	<0.001
mRS at discharge, median (quartiles)	5 (5–6)	4 (3–5)	3 (2–4)	2 (1–4)	<0.001

aPTT indicates activated partial thromboplastin time; DOAC, direct oral anticoagulant; eGFR, estimated glomerular filtration rate; ICA, internal carotid artery; M1, middle cerebral artery horizontal segment; M2, middle cerebral artery insular segment; mRS, modified Rankin Scale; NIHSS, National Institutes of Health stroke scale; PT‐INR, prothrombin time‐international normalized ratio; VKA, vitamin K antagonist.

a Including ischemic stroke and systemic embolism.

b Including ischemic heart disease and peripheral artery disease.

**Table 3:** Result of Multivariate Ordinal Logistic Regression Model for More Proximal Arterial Occlusion

Variables	OR	95% CI	*P* Value
Female sex	1.18	0.74 to 1.88	0.477
Age (per 10 y)	1.36	1.06 to 1.75	0.017
Congestive heart failure	1.56	0.98 to 2.47	0.114
Onset to arrival (per 1 h)	1.00	0.99 to 1.00	0.358
Glucose (per 100 mg/dL)	2.39	1.37 to 4.18	0.002
BNP (per 100 pg/mL)	1.11	1.02 to 1.22	0.017
D‐dimer (per 1 μg/mL)	1.01	0.99 to 1.03	0.305
Anticoagulant status before the event			
No anticoagulant therapy	1.00	Ref.	
Undertreated VKA	1.56	0.82 to 2.99	0.176
Sufficient VKA	0.39	0.16 to 0.98	0.046
DOAC	0.34	0.14 to 0.81	0.015

BNP indicates brain natriuretic peptide; CI, confidence interval; DOAC, direct oral anticoagulant; VKA, vitamin K antagonist.

## Discussion

The present study showed that DOAC medication before the event was related to smaller infarct volume and independently associated with decreased risk of more proximal arterial occlusion in patients with NVAF and ischemic stroke, compared with no anticoagulation or undertreated VKA therapy.

Patients with prior DOAC treatment tended to exhibit smaller infarct volume than those with no anticoagulant therapy, and DOAC medication was independently associated with decreased risk of more proximal arterial occlusion. Although negative associations between VKA therapy and initial stroke volume^9^, ^11^, ^12^ or major artery occlusion^10^ in acute ischemic stroke patients mainly with AF were reported previously, there were few previous studies of the effect of prior DOAC treatment on infarct volume or the site of arterial occlusion. However, the relationship between DOAC therapy and small infarct volume or no proximal artery occlusion seems to be theoretically plausible, because DOAC treatment offers pharmacokinetically consistent and adequate anticoagulation.^13^, ^14^, ^15^, ^16^ Sufficient anticoagulant treatment was known to have an important role in stroke severity or proximal artery occlusion for VKA‐treated AF patients,^23^ and indeed sufficient VKA therapy was also associated with smaller infarct volume or decreased risk of more proximal occlusion in the present study. Small and vulnerable thrombi are expected to be formed under consistent or sufficient anticoagulation. Small thrombi seem to contribute directly to no proximal artery occlusion, and vulnerable thrombi contribute indirectly through ultra‐early recanalization. Lower D‐dimer levels in patients prescribed a DOAC (and patients with sufficient VKA therapy), shown in the present study, may support this hypothesis. DOAC medication before the event should be associated with no major artery occlusion, small infarct volume, and therefore mild symptoms.

There are several limitations to be addressed in the present study. First, nearly identical infarct volume in patients with sufficient VKA therapy and those treated with DOAC suggests the DOAC‐treated patients were compliant. However, anticoagulant status before the stroke was defined based on the prescription information, and duration of anticoagulant therapy or drug adherence information was not systematically collected. This seems to be 1 of the nature of “real‐world” observational study, but adherence was reported to be not high among patients prescribed oral anticoagulants,^24^ and because of the absence of routine anticoagulation monitoring with DOAC treatment, such as PT‐INR with VKA therapy, medication adherence with DOAC treatment could not be estimated. Moreover, the usual indicators of VKA therapy control, such as time in the therapeutic range before stroke, were unavailable. This may underestimate the favorable effect of DOAC treatment on stroke volume or the site of arterial occlusion. Second, because this was a retrospective study from a prospective registry of consecutive patients, the method of detecting paroxysmal AF was not predefined. These may also lead to under‐ and over‐estimation of the effect of DOAC treatment on initial infarct volume or the site of arterial occlusion. Third, because the sample size was relatively small, and the patients included in the present study were old (median age 79 years) and arrived to the hospital within a short time (median 4.5 hours) from onset, the results of the present study may not be generalizable. The present findings should be confirmed with a prospective large cohort and information about medication adherence.

In conclusion, patients with DOAC treatment before the event showed smaller infarct volume, and DOAC treatment was independently associated with decreased risk of more proximal artery occlusion in patients with ischemic stroke and NVAF, compared with no anticoagulation or undertreated VKA therapy.

## Disclosures

None.
